# The impact of difficult embryo transfer on the success of IVF: a systematic review and meta-analysis

**DOI:** 10.1038/s41598-023-49141-x

**Published:** 2023-12-14

**Authors:** Giulia Galati, Marco Reschini, Laura Mensi, Camilla Di Dio, Edgardo Somigliana, Ludovico Muzii

**Affiliations:** 1 Department of Maternal and Child Health and Urology, Sapienza University, 00161 Rome, Italy; 2 Infertility Unit, Fondazione IRCCS Ca’ Granda Ospedale Maggiore Policlinico, Milan, Italy; 3 Department of Clinical Sciences and Community Health, University of Milan, Milan, Italy

**Keywords:** Health care, Medical research

## Abstract

The procedure of embryo transfer (ET) must be as gentle as possible since any traumatism may cause uterine contractility that interferes with the implantation. However, this ideal conduct is not always possible, and additional measures may be necessary (difficult ET). Different studies have evaluated the impact of difficult ET on the clinical pregnancy rate (CPR), but results were not univocal. The present systematic review and meta-analysis was aimed to provide a precise estimate of the possible detrimental effects of difficult ET on CPR. The study protocol was registered online (PROSPERO number: CRD42023387197). An electronic database search was performed to identify articles published until September 2022. The primary outcome was CPR. Fifteen studies fulfilled the inclusion criteria. Difficult ET significantly reduced the CPR (OR 0.70; 95%CI: 0.64–0.76; p < 0.0001. All pre-planned subgroup analyses according to study design (retrospective vs prospective studies), historical period (studies published before and after 2010), type of catheter, frequency of difficult cases (> or < 19%) and pregnancy rate (> or < 38%) confirmed the significant association. Difficult ET is associated with a significant reduction of CPR. Further studies are warranted to understand how to prevent or manage this common clinical situation.

## Introduction

Successful embryo implantation in IVF requires a viable embryo, a receptive endometrium, and a finest technique. The procedure of embryo transfer (ET) is unanimously claimed to be crucial for the success of IVF^1^. The procedure should be guided by ultrasound, soft catheters should be preferred, and the embryos should be released at about 1–2 cm from the fundus of the uterus^1–3^. There is also evidence suggesting that concomitant administration of hyaluronic acid (HA), Granulocyte Colony Stimulating Factor (G-CSF), human Chorionic Gonadotropin (hCG), or atosiban may improve the success rate, but the effectiveness of these add-ons remains debated^1,4^.

Fanchin and Ayoubi affirmed that the procedure of ET must be as gentle as possible since any traumatism may cause the release of oxytocin, boosting the uterine contractility that interferes with the implantation^5^. However, this ideal conduct is not always possible. The insertion of the catheter in the uterine cavity may be difficult because of a stenosis and a tortuosity of the cervical canal or a severe anteversion/retroversion of the uterus. Hard catheters may be needed in these cases. In most challenging situations, the application of tenaculum forceps may be required to allow counter traction and facilitating the insertion of the catheter. All the situations deviating from an unremarkable transfer are grossly unified under the term “difficult ET”.

Different studies have evaluated the impact of difficult ET on the clinical pregnancy rate, but results were inconsistent. However, a meta-analysis performed in 2013 and including only five studies highlighted a RR of clinical pregnancy of 0.75 (95%CI: 0.66–0.86)^6^. We deem of relevance further investigating this issue by adjourning this meta-analysis because a definitive demonstration of a detrimental effect can have immediate clinical implications. One may consider studying more in-depth pharmacological interventions that can temper the uterine contractility or postponing the transfer and freezing of all embryos. This latter approach would allow to tune the best strategy for the insertion of the catheter in a calm context without the need for transferring (proof of transfer or mock ET). To note, some centers systematically perform a preliminary mock ET to identify women at risk of difficult ET and better plan the technique.

## Materials and methods

### Search strategy

The present systematic review and meta-analysis was performed in accordance with guidelines from Cochrane Collaboration and followed Preferred Reporting Items for Systematic Reviews and Meta-Analyses guidelines. The study protocol was registered online in the International Prospective Register Systematic Reviews (PROSPERO number: CRD42023387197). An Ethical Committee acceptance was not requested since only published and anonymized data were used.

An electronic database search was performed to identify articles published until September 2022. Pubmed and Medline were screened to identify studies that evaluated difficult ET and its effects on pregnancy rate using a combination of the following terms: “difficult embryo transfer” OR “easy embryo transfer”. Inclusion criteria of studies were: (1) English language, (2) evaluation of the outcome, (3) patients undergoing fresh or frozen–thawed ET, (4) presence of a control group. After confirmation of pertinence, studies were excluded if reporting partial or incomplete data.

A broadly inclusive search was conducted initially followed by a subsequent restriction for studies focused on pregnancy rate after difficult ET during the title/abstract review process. Data presented exclusively at meetings were not considered.

The reference lists of reviews and relevant articles were screened by hand to identify additional eligible publications. Articles considered were randomized clinical trials (RCTs), prospective or retrospective observational studies. Case reports or small series were excluded. The protocol was designed a priori defining methods for collecting, extracting, and analyzing data. The electronic search was conducted independently by two investigators (G.G. and M.R.). All articles considered relevant based on the title and abstract were retrieved. Subsequently, three investigators (C.D.D., G.G., and L.M.) independently read the full text of the pre-selected articles to verify the pertinence of those for the aim of this analysis. Studies were excluded if reporting partial or duplicate data sets. In case of disagreement on the inclusion or exclusion of preselected studies for meta-analysis, or any other disagreement through the review process, the consensus was reached after discussion involving all researchers.

### Quality assessment

The studies were then classified qualitatively according to the guidelines published in the Cochrane Handbook for Systematic Reviews of Interventions. Two authors (G.G. and L.M.) independently assessed the selected studies for risks of bias using the Newcastle–Ottawa Quality Assessment Scale.

The authors also graded the quality of evidence using the Grading of Recommendations Assessment, Development and Evaluation (GRADE) approach^7^. Quality of evidence was downgraded by one level for serious concerns and by two levels for very serious concerns for risk of bias, inconsistency, indirectness, imprecision, and publication bias.

### Outcomes

The primary outcome was Clinical pregnancy rate (CPR), defined as the presence of an intrauterine gestational sac. The secondary outcome was live birth rate. The following subgroup analyses were pre-planned: mode of recruitment (retrospective or prospective), study period (divided according to the date of publication, before and after 2010), type of catheter (Cook or Wallace catheters), frequency of difficult cases (using the median to divide into two groups) and CPR in the control group (using the median to divide into two groups). If an unpaired number of studies was selected, the study corresponding to the median was included in the group above that median.

The impact of difficult embryo transfer on clinical pregnancy was expressed using the odd ratio (OR) with 95% CI.

The inconsistency of the studies’ results was measured using Cochrane Q and the *I*^2^ statistic^8^. Risk estimates were combined in a meta-analysis using a fixed effects model when the heterogeneity found among the studies was absent to moderate (0% ≤ *I*^2^ < 30%). When heterogeneity was moderate, substantial, or considerable (I^2^ ≥ 30%), the DerSimonian and Laird method was used^9,10^ for a random-effects model. All analyses were performed using Review Manager (RevMan) [Computer program], Version 5.4, The Cochrane Collaboration, 2020.

## Results

### Study selection

**Figure 1:**
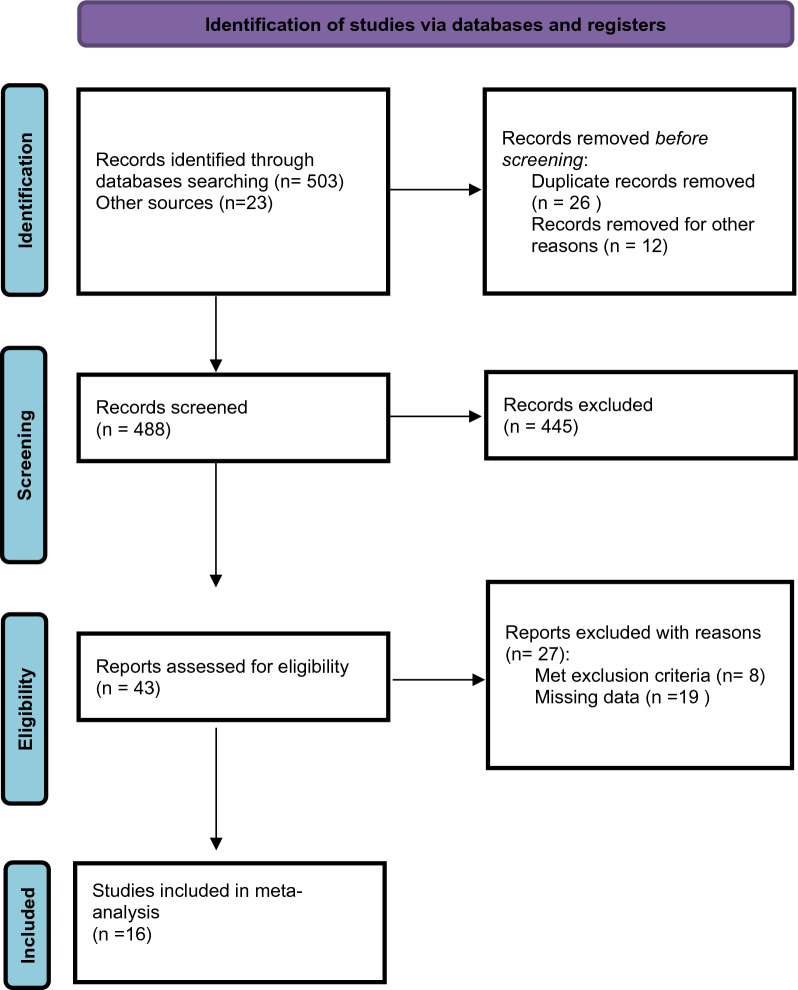
PRISMA flowchart for study identification and inclusion/exclusion.

Our electronic databases search initially yielded 526 articles. Title and abstract screening selected a total of 43 studies eligible for full-text evaluation. Twenty-seven of them were excluded as detailed in the PRISMA flowchart in Fig. 1. Sixteen studies fulfilled the inclusion criteria: their main characteristics are shown in Table 1.

**Table 1:** Characteristics of the included studies.

Author	Year	Study design	Country	Recruitment period	Sample size	Outcomes
Alvarez M	2019	Retrospective observational study	Spain	2014–2016	370	CPR, LBR
Alvero R	2003	Retrospective observational study	USA	1998–1999	583	CPR
Coats E	2019	Prospective cohort study	UK	2014–2016	174	CPR, LBR
Ghaffari F	2013	Prospective cohort study	Iran	2009–2010	706	CPR
Kava-Braverman A	2016	Retrospective observational study	Spain	2009–2015	7611	CPR, LBR
Larue L	2016	Prospective observational study	France	2010–2012	306	CPR
Larue L	2020	Prospective observational study	France	2014–2020	2046	CPR
Listijono DR	2013	Retrospective observational study	Australia	2005–2010	6484	CPR, LBR
Noyes N	1999	Retrospective observational study	USA	1995–1998	1117	CPR
Plowden TC	2017	Retrospective observational study	USA	2012–2013	922	CPR, LBR
Öztürk İnal Z	2021	Prospective cohort study	Turkey	2012–2017	2257	CPR
Singh N	2012	Retrospective observational study	India	2008–2010	342	CPR
Spitzer D	2012	Retrospective observational study	Austria	2005–2009	1055	CPR, LBR
Tomás C	2002	Retrospective observational study	Finland	1994–2000	4807	CPR
Tur-Kaspa I	1998	Retrospective observational study	Israel	1994–1996	854	CPR, LBR
Yilmaz N	2013	Retrospective observational study	Turkey	2010–2012	313	CPR

CPR, clinical pregnanct rate; LBR, live birth rate.

### Study characteristics

All 16 studies were included in the meta-analysis encompassing a total of 29,947 patients^11–26^. The ET was quoted as difficult in 3544 cases (11.83%). Five were prospective controlled studies and eleven were retrospective studies. All studies reported the clinical pregnancy rate. Five studies also reported miscarriage rate and only seven reported the live birth rate. The definition of difficult embryo transfer was very similar. All but one defined the difficulty as the need for additional measures (use of hard catheter, malleable stylet, or tenaculum). Only one study referred to the subjective assessment of the operators^13^. Three studies performed ET using Cook catheter and seven using Wallace catheter. In four studies the enrolment period ranged from 1998 to 2005, in twelve studies it ranged from 2016 to 2017.

### Risk of bias and quality assessment results

Results obtained from our risk of bias assessment for all the 16 studies are summarized in Supplemental Table 1. Overall, the quality assessment showed low risk of bias. Among the nine applicable stars assessing three main categories of selection, comparability and outcomes, the eligible studies received eight.

The quality of evidence according to the GRADE system was low in all studies.

### Results of syntheses

**Figure 2:**
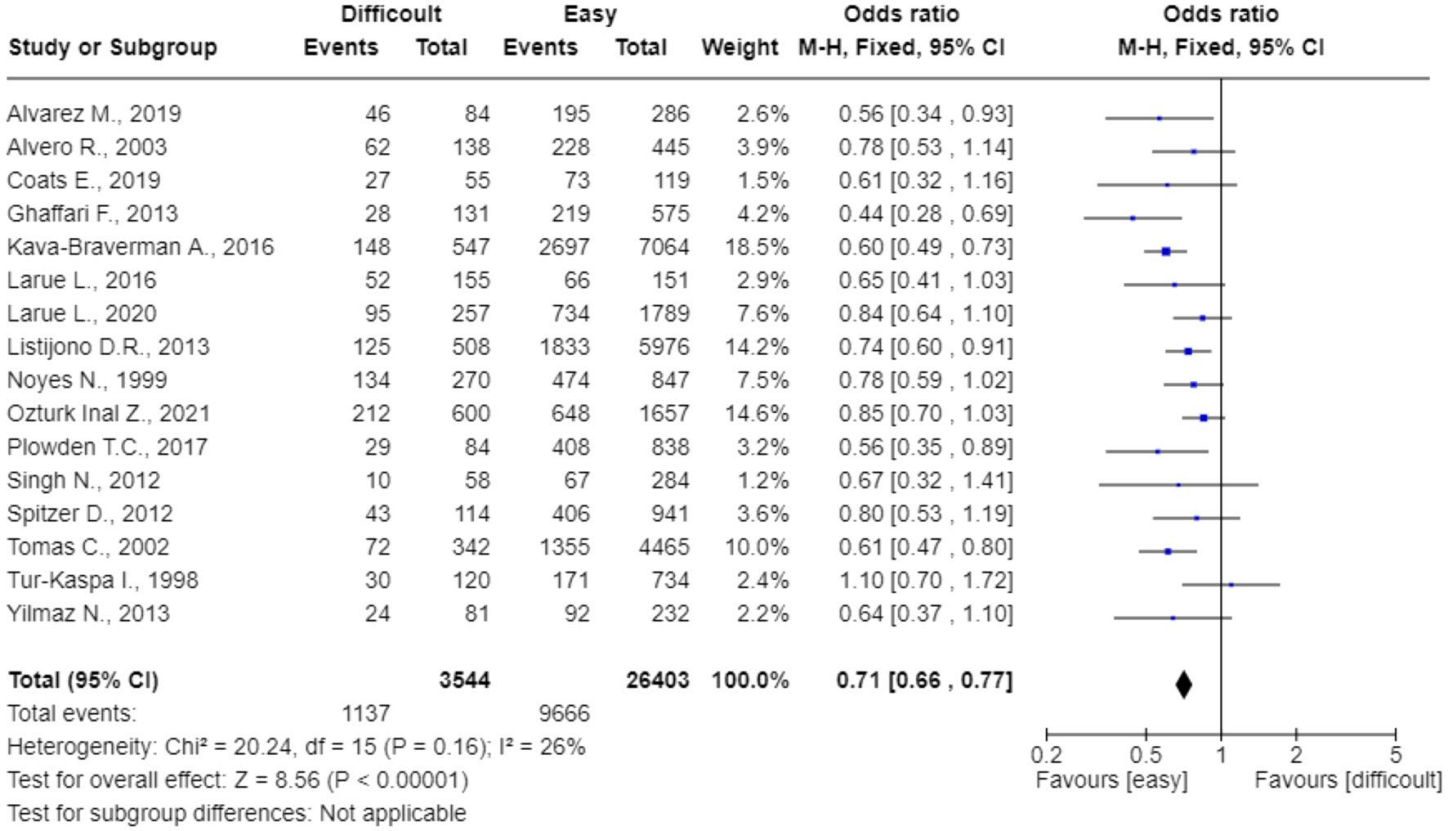
The effect of Difficult Embryo Transfer on the clinical pregnancy rate.

**Figure 3:**
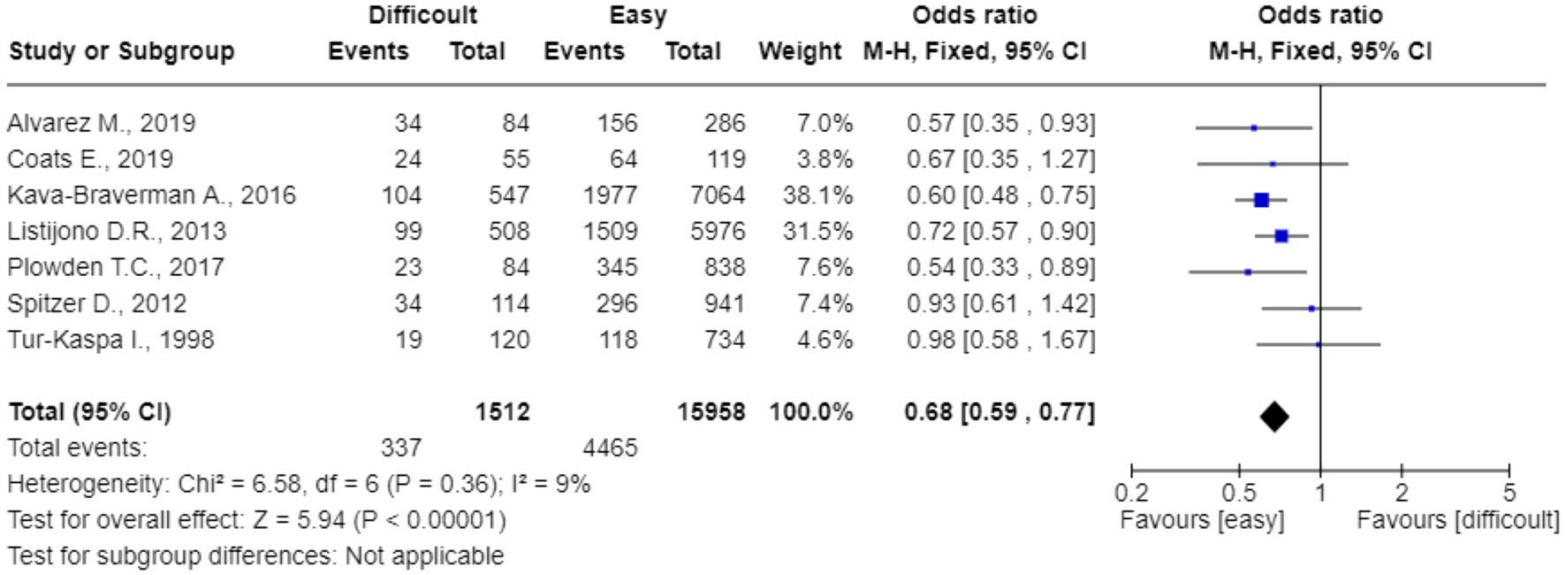
The effect of Difficult Embryo Transfer on the live birth rate.

All 16 studies including women who performed ET were meta-analyzed^11–26^. Difficult ET group significantly reduced the pregnancy rate. The pooled OR was 0.71 (95%CI: 0.66–0.77; p < 0.0001) (Fig. 2). Heterogeneity for this comparison was I^2^ 26%. When focusing on live birth rate (n = 7 studies), the pooled OR was 0.68 (95%CI: 0.59–0.77; p = 0.00001) and the heterogeneity was I^2^ 9% (Fig. 3).

**Figure 4:**
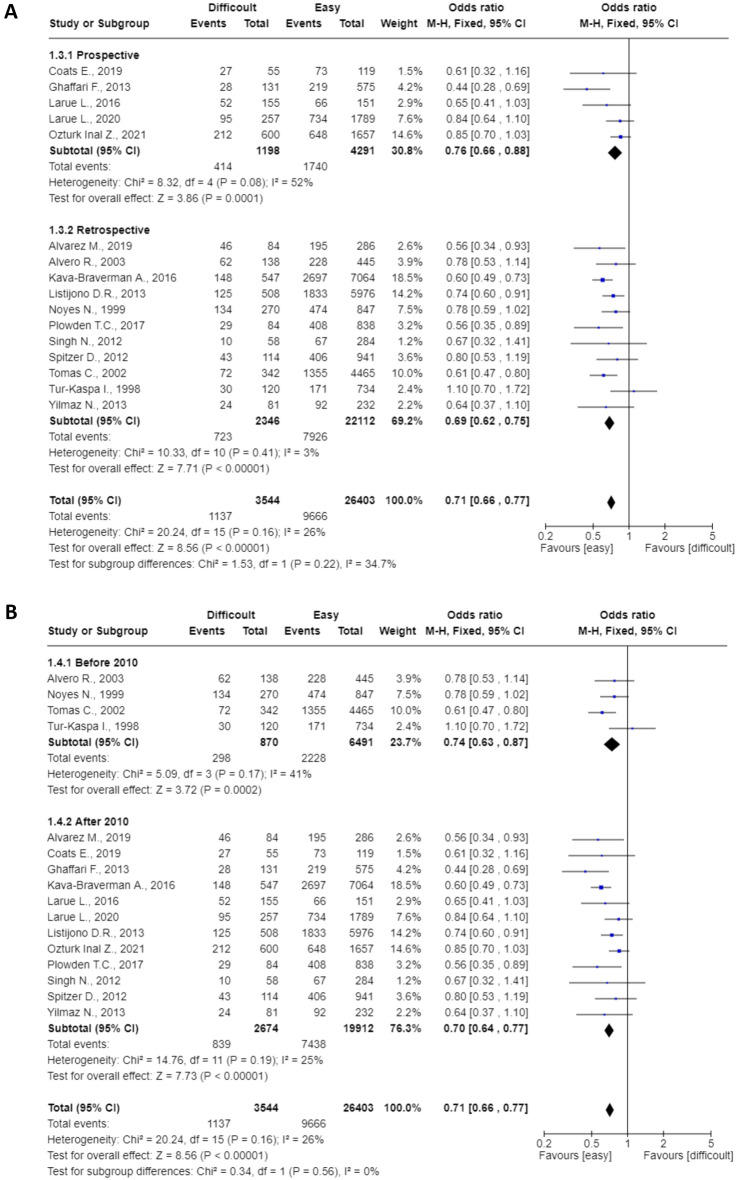

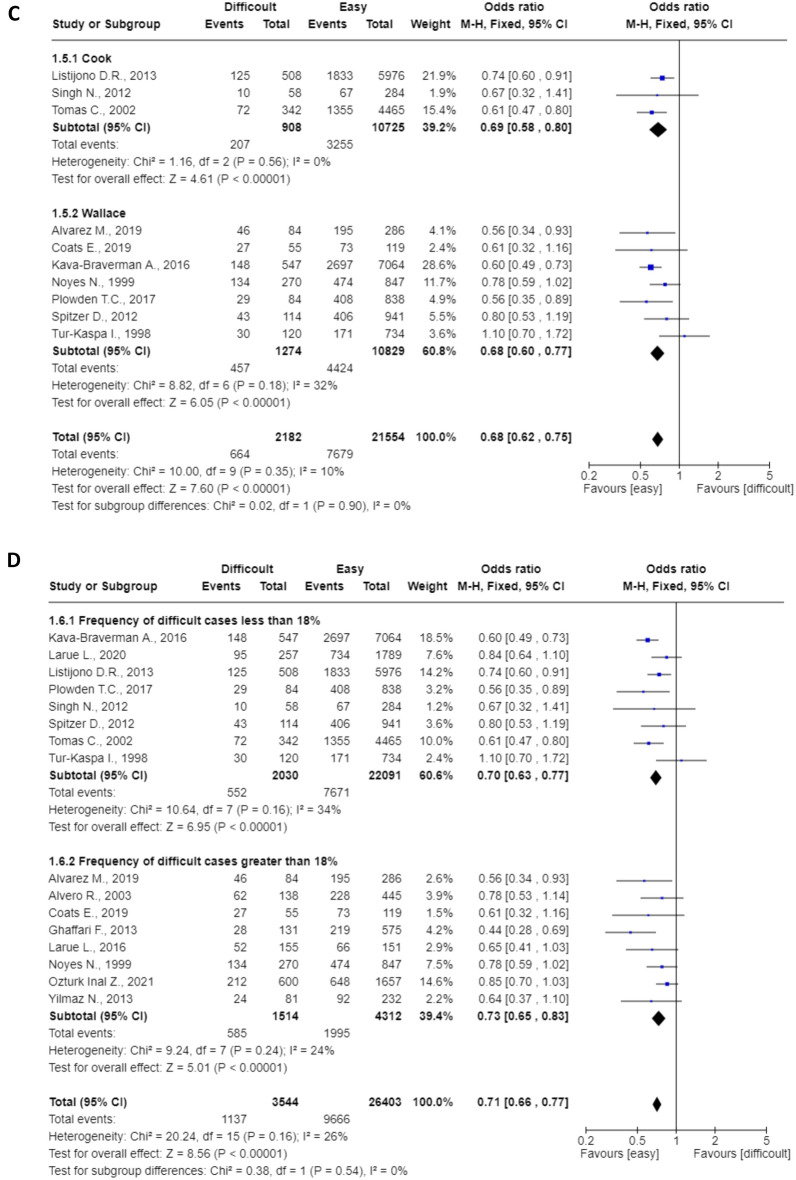

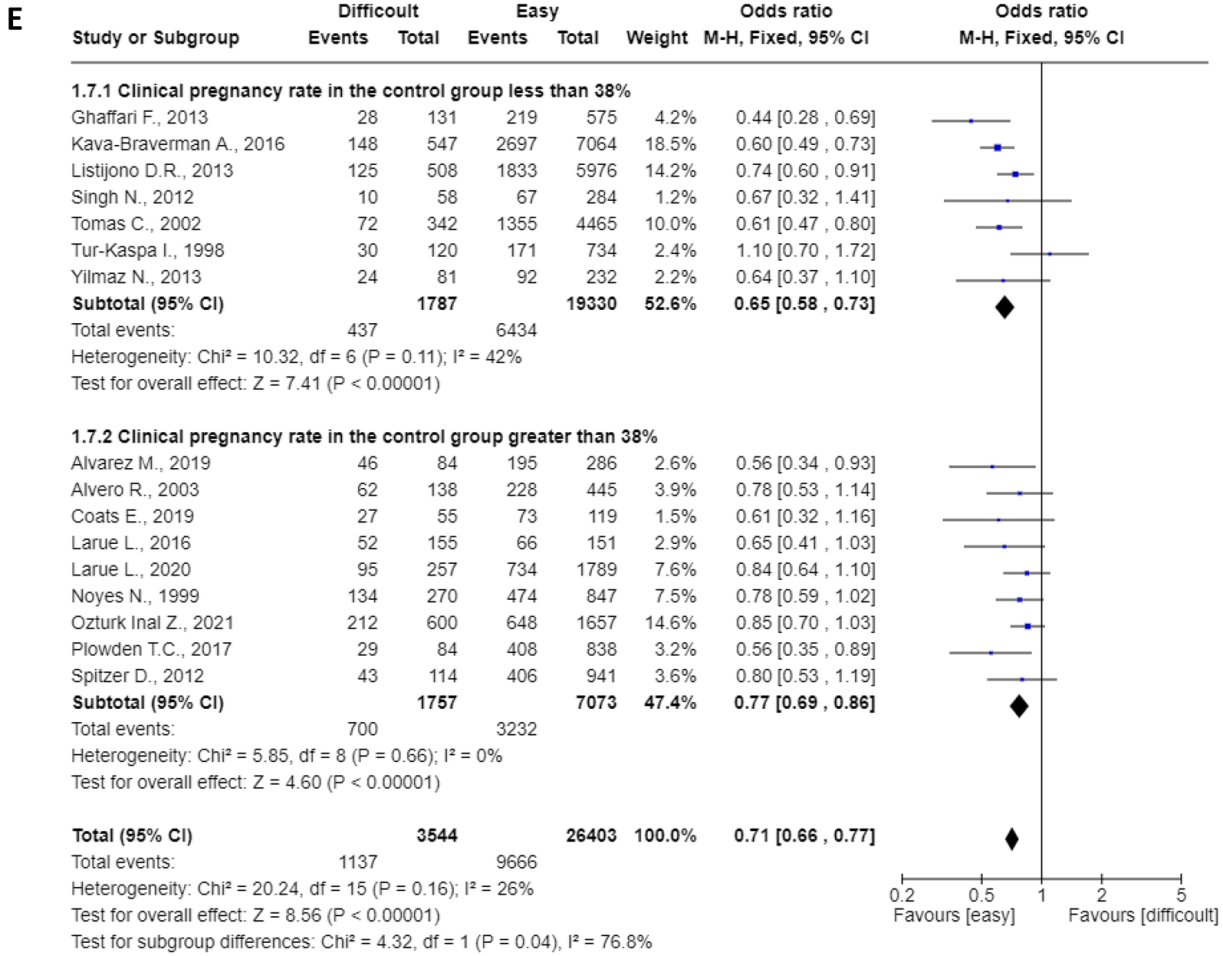
(**A**) mode of recruitment (retrospective or prospective); (**B**) study period (divided according to the date of publication, before and after 2010); (**C**) type of catheter (Cook or Wallace catheters); (**D**) frequency of difficult cases (using the median to divide into two groups); (**E**) CPR in the control group (using the median to divide into two groups).

Pre-planned subgroup analyses are shown in Fig. 4. All were statistically significant (p < 0.0001) and heterogeneity varied between 0 and 42%. The pooled OR for prospective^14–17, 21^ and retrospective studies^11–13, 18–20, 22–26^ were 0.76 (95%CI: 0.66–0.88) and 0.69 (95%CI: 0.62–0.75), respectively (Fig. 4A). The pooled OR for studies published before (n = 4) and after (n = 12) 2010 were 0.74 (95%CI: 0.63–0.87) and 0.70 (95%CI: 0.64–0.77), respectively (Fig. 4B). The pooled OR for studies in which the ET was performed using Cook (n = 3) or Wallace (n = 7) catheters were 0.69 (95%CI: 0.58–0.80) and 0.68 (95%CI: 0.60–0.77), respectively (Fig. 4C). The pooled OR for studies with a frequency of difficult cases greater (n = 8) and less (n = 8) than 18% (cut-off chosen based on the median) were 0.73 (95%CI: 0.65–0.83) and 0.70 (95%CI: 0.63–0.77), respectively (Fig. 4D). The pooled OR for studies with clinical pregnancy rate in the control group greater (n = 9) and less (n = 7) than 38% (cut-off chosen based on the median) were 0.77 (95%CI: 0.69–0.86) and 0.65 (95%CI: 0.58–0.73), respectively (Fig. 4E).

## Discussion

The present meta-analysis confirmed previous findings suggesting that difficult ET was associated with a significant reduction in the chance of clinical pregnancy^6^. The estimated impact is a relative reduction of 30%, a rate that is clinically remarkable and relevant. To note, the low amplitude of the 95%CI (0.64–0.76) allows us to infer that the relative reduction is at least 24%. In other words, difficult ET is associated to the wastage of a viable embryo able to implant in at least one in four women.

The previous meta-analysis published in 2013 included only five studies (the estimate was inevitably less precise), the authors could not thus perform subgroup analyses and results inevitably referred to older series^6^. Our results extend the findings by reporting a more precise estimate of the detrimental effect (we included 16 studies) and by highlighting that the progresses in IVF instrumentations have not overcome the issue. Results were similar regardless of the type of catheter used and, most importantly, the historical subgroup analyses (i.e., when we evaluated separately studies published before and after 2010) did not highlight any change over time. There is even a slight trend to a more relevant impact in more recent studies (OR = 0.68, 95%CI: 0.62–0.75).

The additional subgroup analyses always confirmed the significant association of difficult ET with reduced clinical pregnancy rate. We failed to identify any subgroup that did not show this effect. Of interest is the observation that the effect is independent from the local rate of success and the frequency of difficult ET. The negative effect was identified in both Centers with higher and lower rate of success and in those with higher and lower rate of difficult ET. Based on this latter finding, one is tempted to speculate that the expertise of the team (that may be reflected by a lower rate of difficult ET) may ultimately have clinically relevant benefits. To note, there is evidence from intra-group comparisons that the rate of success may differ among physicians of the same center^27^.

The main objective of the ET is to gently deposit the embryos inside the uterine cavity and in a correct position to maximize the chance of implantation. The American Society for Reproductive Medicine (ASRM) guidelines describe better pregnancy rates with several measures, such as the use of abdominal ultrasound during the transfer, the removal of cervical mucus, the use of a soft catheter and the deposition of the embryo at about 1–2 cm from the uterine fundus^2^. Recently, D’Angelo et al.^3^ provided for ESHRE (European Society of Human Reproduction and Embryology) an overview of technical aspects of ET, including the preparation prior to ET, the procedure itself, the post-procedure care, and the importance of the physicians’ performance. Their recommendation overall overlap with those of the ASRM. Surprisingly, both papers do not deal with the situation of difficult ET. In most cases, this situation is unexpected and the physician performing the transfer must contravene to the dogma of gentleness. He may need hard catheter or must use a tenaculum. No recommendation is given to guide the physician in this common situation. This is surprising since at least two approaches may deserve attention. First, one may consider oxytocin receptor antagonists that can temper uterine contractions. There is evidence that atosiban or other agents of the same family may increase the success rate of ET, in particular in women with repeated implantation failure^1,28^. However, the possibility to use this drug exclusively in cases of difficult embryo transfer, after the difficult but definitive insertion of the catheter but prior to the release of the embryos has not been investigated. In our opinion, this approach may be rational and deserves investigation. Second, one may consider suspending the transfer, freezing the embryos, and postponing the procedure to the following month (transforming the transfer in a mock transfer). This is harsher for the women and may require to re-freeze embryos that were just thawed. However, it allows the following month to be more prepared, involving most expert physicians, and knowing in advance the possible difficulties, the trajectory that the catheter should follow, and the most suitable catheter to use. Randomized Controlled Trial are required, but the stake is clinically valuable, considering that these interventions may potentially increase in the rate of success in this population with an OR of 1.4 (1/0.7). The scant attention given to this topic is surprising.

Some strengths and limitations of this meta-analyses deserve to be recognized and discussed. Considering the former, this is the largest systematic review and meta-analysis on the impact of difficult embryo transfer on reproductive outcomes. The previous one included one third of the papers selected for the present one (5 compared to 15). Moreover, evidence is rather consistent, and heterogeneity is generally limited, supporting the robustness of our conclusions.

On the other hand, some limitations should be acknowledged. First, the criteria used for defining difficult ET are not precise. Most refers to the need for additional measures, a pragmatic but imprecise and poorly reproducible definition. Not surprisingly, despite a similar theoretical definition (“needs for additional measures”), the rate markedly differed among selected studies, varying between 7 and 32%. A second limitation is that a considerable portion of the data is from only two studies^12,18^. However, when excluding these two studies, the significant association remained, and the magnitude was similar (data not shown). Third, the quality of the available evidence is not optimal. Even if the Newcastle–Ottawa Quality Assessment Scale quoted well all the included studies, none of them reached the standards to be defined of moderate-high quality according to the GRADE system. Moreover, data on live birth rate (the gold standard for studies on IVF) was available in less than half of the studies. However, given the specific query of this study (implantation of the embryo), we do not deem this limit of major relevance and, therefore, we decided a priori for clinical pregnancy rate rather than live birth rate as a primary outcome. Fourth, the clinical policies of the different studies varied widely. Of utmost relevance here is the inclusion of fresh and frozen cycles and, among the latter, of both women treated with hormone replacement therapy and natural cycles. We cannot exclude that the impact of difficult ET on the rate of success may vary according to the clinical conditions associated to the transfer. We could not assess this important aspect in our meta-analysis. Finally, our observational design does not allow us to infer a causal relation between the need for additional maneuvers and the chance of success. We cannot exclude that the anatomical peculiarities making the transfer more difficult may also impair on their own the implantation of the embryo. An RCT comparing gentle and aggressive techniques of transfer would be necessary to provide a definitive answer, but this type of study would be ethically debatable.

In conclusion, our study indirectly supports the idea that the ET should be done gently, trying to avoid the use of additional instruments that can facilitate the access to the endometrial cavity but can concomitantly affect the implantation of the embryo. Given the importance of the ET as the final step of the IVF cycles, every effort should be made to optimize the procedure, to predict and reduce the likelihood of encountering difficulties and to overcome the detrimental secondary effects in case of a difficult access. Further studies should be designed to achieve this crucial goal that may allow to significantly improve IVF outcomes.

### Supplementary Information

Supplementary Table 1.

## Data Availability

The datasets generated during and/or analysed during the current study are available from the corresponding author on reasonable request.
