# Supplementary material for: The impact of difficult embryo transfer on the success of IVF: a systematic review and meta-analysis
Source: Sci Rep. 2023 Dec 14;13:22188. doi: 10.1038/s41598-023-49141-x (PMC10719337; doi:10.1038/s41598-023-49141-x)
Supplement: Supplementary file 1 — Supplementary Table 1. [file 41598_2023_49141_MOESM1_ESM.docx]

| **Supplemental Table 1: Risk of bias assessed assessed by the Newcastle-Ottawa Scale (NOS) for observational and nonrandomized studies.** | | | | | | | | | | |
| --- | --- | --- | --- | --- | --- | --- | --- | --- | --- | --- |
|  |  |  |  |  |  |  |  |  |  |  |
| **Author** | **Year** | **Selection** | | | | **Comparability** | **Outcome** | | | **Total score** |
|  |  | **Representativeness of the exposed cohort** | **Selection of the non-exposed cohort** | **Ascertainment of exposure** | **Outcome of interest was not present at the start of the study** | **Comparability of cohorts on the basis of the design or analysis** | **Assessment of outcome** | **Was follow-up long enough for outcomes to occur?** | **Adequacy of follow up of cohorts** |  |
| Öztürk İnal Z | 2021 | * | * | * | * | * | * | * | * | 8 |
| Alvarez M | 2019 | * | * | * | * | * | * | * | * | 8 |
| Kava-Braverman A | 2016 | * | * | * | * | * | * | * | * | 8 |
| Plowden TC | 2017 | * | * | * | * | * | * | * | * | 8 |
| Ghaffari F | 2013 | * | * | * | * | * | * | * | * | 8 |
| Listijono DR | 2013 | * | * | * | * | * | * | * | * | 8 |
| Singh N | 2012 | * | * | * | * | * | * | * | * | 8 |
| Tomás C | 2002 | * | * | * | * | * | * | * | * | 8 |
| Noyes N | 1999 | * | * | * | * | * | * | * | * | 8 |
| Tur-Kaspa I | 1998 | * | * | * | * | * | * | * | * | 8 |
| Coats E | 2019 | * | * | * | * | * | * | * | * | 8 |
| Alvero R | 2003 | * | * | * | * | * | * | * | * | 8 |
| Spitzer D | 2012 | * | * | * | * | * | * | * | * | 8 |
| Larue L | 2016 | * | * | * | * | * | * | * | * | 8 |
| Larue L | 2020 | * | * | * | * | * | * | * | * | 8 |
| Yilmaz N | 2013 | * | * | * | * | * | * | * | * | 8 |
